# ‘Techtorial’: Changing paradigms

**DOI:** 10.4102/sajr.v26i1.2571

**Published:** 2022-12-19

**Authors:** Maya Patel

**Affiliations:** 1 Department of Radiology, School of Medicine, Faculty of Health Sciences, University of the Witwatersrand, Johannesburg, South Africa; 2 National Radiology Services Inc, Johannesburg, South Africa

Long gone are the days of the film library and the tedious grind through packets of radiographs, heavy-weighted textbooks and stacks of printed journal articles. Thanks to technology, there has been a revolutionalised approach to the way we learn and teach radiology. New generation radiologists are fully immersed in the realm of the Internet where they self-learn remotely through online resource sites, courses, blogs and social media. Trainees are also able to perform self-evaluation tasks, have imaging reports reviewed and attempt examination preparation scenarios. Such services offer immediate gratification, convenient availability at any time or place, and the ability to learn from different institutions and experts from anywhere in the world. Spurred on by the recent pandemic, when in-person teaching interaction was interrupted, new avenues for learning have sprouted and remain popular despite the return to ‘normality’.

So, is medical journalism also veering off course? Interestingly, apart from conversion to a digital platform, most journals still function like before, continuing in their role as the cornerstones of academia. On average, a research manuscript requires years of data acquisition, 3–6 months for data analysis and write-up, and another 6–9 months of reviewing, processing and editing by journal publishers before the reward of submission acceptance and printing. This painstaking process is undertaken with the aim of sharing derived findings and knowledge with the medical community. The information is weighted by statistics and matched with other research, deserving of attention.

Extrapolating from the *SA Journal of Radiology* (SAJR), it is clear that readership has steadily increased over the past five years, but abstract views outnumber the full manuscript pdfs. Trends seen in international journals suggest that this is probably not a unique phenomenon. In an attempt to improve dissemination of information, medical journalism too has taken to social media and blogs. Authors are recording videos of themselves delivering their manuscript abstracts and academic journals are even being converted to audible formats. Is this the way forward?

Our brains process an extraordinary amount of digital data on a daily basis and journalism needs to burrow its way into this data to reach more audiences. Most radiologists would preferably opt to view a webinar or browse for a summarised chart on updated guidelines, rather than read the published article. Similarly, interesting imaging findings are no longer published as case reports, but rather shared on teaching sites with a few citations or instantaneously communicated via social media. A brief title or image can easily be used to spark interest and persuade further reading via embedded links. As long as the research reaches the intended audience, probably no harm is incurred.

While gaining experience from hands-on learning is ideal and well-referenced academic publishing is the preferred means of sharing knowledge with peers, technology has become intertwined and inseparable from medicine. Embracing the use of alternative multifaceted avenues for dissemination of information seems to be a necessary paradigm adaptation as the world orbits with electronics and our brains favour a digitised landscape.

‘*Progress is impossible without change and those who cannot change their minds cannot change anything*’ (George Bernard Shaw)

